# Case Report: MIS-C With Prominent Hepatic and Pancreatic Involvement in a Vaccinated Adolescent – A Critical Reasoning

**DOI:** 10.3389/fped.2022.896903

**Published:** 2022-05-16

**Authors:** Rita Consolini, Giorgio Costagliola, Erika Spada, Piero Colombatto, Alessandro Orsini, Alice Bonuccelli, Maurizia R. Brunetto, Diego G. Peroni

**Affiliations:** ^1^Section of Clinical and Laboratory Immunology, Division of Pediatrics, Department of Clinical and Experimental Medicine, University of Pisa, Pisa, Italy; ^2^Division of Pediatrics, Department of Clinical and Experimental Medicine, University of Pisa, Pisa, Italy; ^3^Division of Hepatology, Department of Clinical and Experimental Medicine, University of Pisa, Pisa, Italy

**Keywords:** children, COVID-19, SARS-CoV-2, vaccination, hemophagocytic lymphohistiocytosis (HLH)

## Abstract

Multisystem inflammatory syndrome in children (MIS-C) is a pathologic condition that has emerged during the coronavirus disease 2019 (COVID-19) pandemic. Although the epidemiological evidence of association between MIS-C and SARS-CoV-2 infection has been demonstrated, its pathogenic mechanism is still undefined. We describe the case of a 17-year old female, previously vaccinated against SARS-CoV-2, presenting with a history of asthenia, fever, cough, anorexia, abdominal pain, and vomiting. During the hospitalization, the patient developed bilateral conjunctivitis, hand vasculitis, cutaneous rash, and multiple pulmonary nodules, following by hepatitis and pancreatitis. As she reported a high-risk contact with a SARS-CoV-2 positive patient 10 days before admission, the epidemiological link and the clinical picture characterized by multi-system organ disfunction and inflammatory biomarkers increase led us to the diagnosis of MIS-C. Therefore, the patient was treated with intravenous immunoglobulin and corticosteroids, resulting in a rapid resolution of fever, cutaneous, and pulmonary involvement, while the recovery of hepatitis and pancreatitis was observed in the following weeks. This case leads to the discussion on whether SARS-CoV-2 immunized children and adolescents should be considered at risk of developing MIS-C and on their possible presentation with non-classic clinical features. Additionally, due to the increasing number of vaccinated children and adolescents, the issues resulting either from the diagnostic suspect of MIS-C or from the consequent need of an early therapeutic approach are discussed.

## Introduction

Since the first stages of the coronavirus disease 2019 (COVID-19) pandemic, the emergence of a new, distinct phenotype of hyperinflammatory syndrome in children was described. This condition, defined as multisystem inflammatory syndrome in children (MIS-C) (1), has been found to have a direct epidemiological correlation with the spreading of SARS-CoV-2, although its pathogenesis is still not completely elucidated (2). The manifestations of MIS-C range from Kawasaki disease (KD)-like presentations to severe multiorgan involvement leading to circulatory shock and disseminated intravascular coagulation (3, 4). Currently, the criteria used to define MIS-C in clinical practice require the demonstration of an epidemiological correlation between the clinical picture and ongoing or previous interaction with SARS-CoV-2 (5) in a patient presenting with fever, elevation of the inflammatory markers, and a clinical picture of multisystemic involvement. To formulate the diagnosis of MIS-C a positive swab for SARS-CoV-2, a positive serologic testing for anti-SARS-CoV-2 antibodies, or a known high-risk contact with a SARS-CoV-2 positive patient is necessary. The availability of anti-SARS-CoV-2 vaccines in adolescents and children will significantly contribute to the healthcare response against this pandemic, and hopefully reduce also the incidence of MIS-C. Indeed, recent, large cohort studies evidenced that the incidence of MIS-C is remarkably lower in vaccinated people, although the impact of the new viral variants ant the time at which protection is conferred have not been investigated (6, 7).

In this article, we describe the case of an adolescent vaccinated against SARS-CoV-2 and presenting with a systemic clinical picture featured by fever and cutaneous, pulmonary, hepatic, and pancreatic involvement, that was diagnosed with MIS-C following current diagnostic criteria. The analysis of this case leads to the discussion on whether immunized children and adolescents should be considered at risk of developing MIS-C and their potential presentation with atypical disease phenotypes. Moreover, the need to critically revise the diagnostic criteria for MIS-C considering the increasing number of seropositive children in the general population is discussed.

## Case Presentation

**FIGURE 1:**
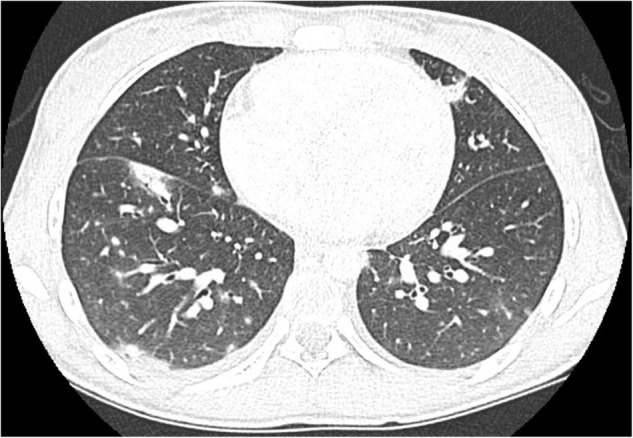
Chest CT at diagnosis of MIS-C showing multiple bilateral pulmonary nodules mainly localized in the basal segments.

**FIGURE 2:**
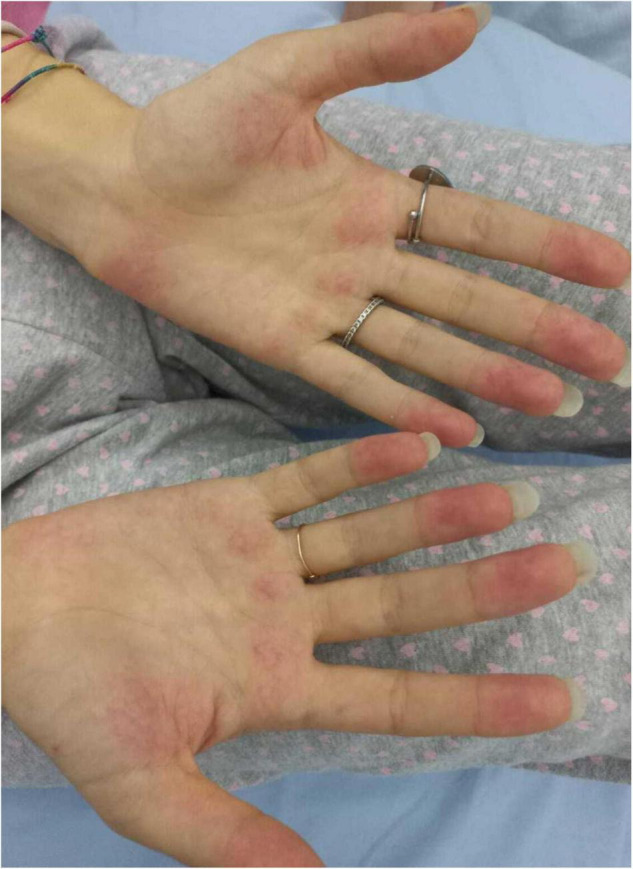
Acral hand vasculitis in our patient.

We herein describe a 17-year old female hospitalized for a history of fever, asthenia, cough, anorexia, abdominal pain, and vomiting. The patient has a positive familial history for autoimmune disorders (inflammatory bowel diseases, connective tissue diseases, and multiple sclerosis). She had paucisymptomatic SARS-CoV-2 infection approximately 1 year before this episode, and was vaccinated with two doses of anti-SARS-CoV-2 mRNA vaccine (Comirnaty), performed 5 and 4 months before hospitalization, respectively, and resulting in a detectable immune response (anti-spike protein antibodies 2,139.6 U/mL, reference value <50 U/mL). She had a high-risk contact (more than 6 h, without protective face mask) with a SARS-CoV-2 positive patients 10 days before hospitalization. At admission, mild splenomegaly was evidenced and laboratory exams showed neutrophilic leukocytosis, elevation of inflammatory markers (erythrocyte sedimentation rate 85 mm/h, reference value <25, C-reactive protein 8.35 mg/dL, and reference value <0.5), transaminases, total and direct bilirubin, and a nasal swab for SARS-CoV-2 identification resulted negative. Chest *X*-ray and abdomen ultrasound resulted normal. During the following days, the fever persisted and she developed bilateral, non-exudative conjunctivitis, cutaneous erythematosus rash at lower limbs and mild hepatomegaly associated with the worsening of abdominal pain, mostly localized in right hypochondrium. Laboratory examinations showed progressive leukopenia (Table 1), worsening of hypertransaminasemia and hyperbilirubinemia, progressive reduction of serum albumin levels, and elevation of D-dimer. Markers of hemophagocytic lymphohistiocytosis (HLH)/macrophage activation syndrome (MAS) were also measured, including ferritin, lactate dehydrogenase, triglycerides, resulting not suitable with this condition. Particularly, the highest ferritin value was 419 μg/L (reference value 30–400), and triglycerides, fibrinogen, and LDH values were within the normal range [triglycerides 180 mg/dL (reference value <200), fibrinogen 308 mg/dL (reference value 200–400), and LDH 151 U/L (reference value <214)]. Microbiological work-up included blood, urine and stool bacterial cultures, and viral serologies and PCR for pathogens associated with infectious hepatitis (Epstein-Barr virus, Cytomegalovirus, Hepatitis A, B, and C virus). Serologic testing for autoimmune hepatitis, cholangitis, and systemic connective tissue diseases were also performed and resulted negative, with the exception of a positivity for antinucleous antibodies (1:160). A peripheral blood smear resulted negative for the presence of schistocytes or atypical cells, and a first-level immunological assessment (serum immunoglobulin and subclasses, lymphocyte subpopulation) did not evidence findings suggestive for an immunodeficiency. To investigate the worsening abdominal pain, a computed tomography (CT) was performed, evidencing hepatomegaly and mild edema in the gallbladder wall, in absence of biliary obstruction. A chest CT showed multiple nodular areas involving both the lungs, and mostly represented in the basal segments (Figure 1). Also, mild pericardial effusion was evidenced. Echocardiography did not evidence anatomical or functional abnormalities, and serum troponin and BNP resulted normal. Meanwhile, the general conditions of the patient continued worsening, and she also presented a hand acral vasculitis (Figure 2) that spontaneously resolved after 24 h. On the basis of the clinical picture of multisystemic involvement (fever, hepato-splenomegaly, hypoalbuminemia, pulmonary nodular involvement, conjunctivitis, erythematous rash, acral vasculitis, and raised inflammatory markers) and the history of recent exposure to SARS-CoV-2, the diagnosis of MIS-C was posed, according to the World Health Organization (WHO) and center for disease control and prevention (CDC) diagnostic criteria (5). Consequently, the patient was treated with intravenous immunoglobulin (IVIG) at a cumulative dose of 2 g/Kg divided into five administrations and accompanied by oral prednisone 1 mg/Kg daily and subcutaneous heparin 100 U/Kg daily. The introduction of treatment was rapidly followed by the resolution of fever, improvement of the general condition, and normalization of the inflammatory markers, while transaminases continued to increase. A week after the introduction of treatment, she presented a relapse of the abdominal pain, associated with elevation of amylase and lipase, and the patient underwent a chest and abdomen CT, that showed a marked improvement of the pulmonary radiologic findings and features consistent with hepatitis and pancreatitis. In the following days, she continued the corticosteroid therapy and received parenteral nutrition for 2 days, resulting in a progressive resolution of the clinical and laboratory picture. The diagnostic work-up was completed by abdomen magnetic resonance which excluded the presence of cholelithiasis and showed a moderate peri-portal inflammatory infiltrate without signs of bile ducts damage. After 30 days of hospitalization, the patient was discharged in good general conditions, without clinical and laboratory evidence of organ involvement, and negative inflammatory markers.

**TABLE 1:** Laboratory assessment at disease presentation and during follow-up.

	Diagnosis of MIS-C	7 days	14 days	21 days	Reference range
Hemoglobin (g/dL)	10.6	12.5	10.5	10.5	12.0–15.0
White blood cells (cells/mm^3^)	14,010	17,280	9,430	8,340	4,000–13,000
Platelets (cells/mm^3^)	228,000	423,000	231,000	225,000	140,000–450,000
Neutrophils (cells/mm^3^)	11,090	14,470	5,890	4,540	1,400–9,100
Lymphocytes (cells/mm^3^)	1,650	1,450	2,470	2,720	800–7,800
ALT (U/L)	159	453	429	367	<40
AST (U/L)	103	145	101	106	<40
γ-GT (U/L)	365	508	454	204	<36
Total bilirubin (mg/dL)	6.59	5.63	5.42	3.43	<1.2
Direct bilirubin (mg/dL)	5.66	4.41	4.52	2.50	<0.3
Amylase (U/L)	/	2,263	99	79	28–100
Lipase (U/L)	/	2,102	100	95	<60
Albumin (g/dL)	3	3.1	3.7	/	>3.5
D-Dimer (μg/mL)	788	3,831	595	434	<500

*ALT, alanine aminotransferase; AST, aspartate aminotransferase; γ-GT, γ-glutamyl transferase.*

## Discussion

The described case of MIS-C in an adolescent who have been vaccinated against SARS-CoV-2 required a critical reasoning in the diagnostic work-up while offering several points for discussion. MIS-C develops in the context of a probable or ascertained SARS-CoV-2 infection, but other possible etiologies should be ruled out. Therefore, our initial diagnostic approach was directed to the identifications of rheumatologic diseases and hyperinflammatory conditions such as KD, HLH, and MAS, overlapping in clinical and laboratory features. The older age compared to patients with KD, together with some laboratory parameters (above mentioned) allowed us to exclude these diagnoses. Furthermore, the association with fever, multiorgan dysfunction, and elevated acute phase reactants, suitable with an underlying systemic hyperinflammation, and the history of SARS-CoV-2 exposure led us to establish the diagnosis of MIS-C.

This case raises the question about the possibility to diagnose MIS-C in vaccinated patients when case definition criteria are suitable. At first, it is important to underline that the response to vaccination, although effective on large-scale populations, is not protective against infection (and, potentially, MIS-C) in a small percentage of patients, that is higher in immunocompromised patients (8) and could increase with the spreading of new virus variants. Noteworthy, it has been recently demonstrated that MIS-C patients display a restricted antibody response, largely limited to anti-spike antibodies with the overall lowest neutralizing activity and lack of anti-nucleocapsid (anti-N) antibodies, suggesting a weaker immune response, almost not neutralizing (9). Additionally, it has been reported that patients with MIS-C carry higher anti-spike antibodies, compared to children infected by SARS-CoV-2 but not developing MIS-C (10).

Moreover, although MIS-C seems a post-infectious severe event arising from a dysregulated immune response leading to a hyperinflammatory state, its pathogenesis is not completely elucidated. Different theories have been formulated to explain its outbreak during the pandemic, including the role of molecular mimicry, superantigen-dependent immune activation, delayed interferon response, presence of anti-interferon autoantibodies, dysregulation of the inflammasome by an extensive innate immune response primed by Toll-like Receptors expressed on ACE + type II pneumocytes, antibody-dependent enhancement, and others (11–13). Specific molecular signatures have been evidenced in MIS-C patients, highlighting the role of type II interferon and NF-κB activation in its pathogenesis (14). The analysis of T-cell receptor repertoire, showing an expansion of TCRβ variable gene 11-2 (TRBV11-2) that correlates with disease severity and serum cytokine levels, suggests the contribution of a superantigen-dependent activation of T cells in the pathogenic process leading to MIS-C (14–16). Additionally, the role of the individual genetic background has been suggested, as the development of MIS-C is associated with distinct HLA alleles (14). These aspects, and specially the involvement of the innate immune response and the superantigen-dependent T-cell activation, are consistent with the need to consider MIS-C even in immunized patients.

To our knowledge, only rare cases of multisystem inflammatory syndrome directly triggered by SARS-CoV-2 vaccination have been reported in adults and adolescents (13, 17–19). Also the reports of classical case of MIS-C (triggered by SARS-CoV-2 exposure) in vaccinated patients are of extreme rarity. A study by Zambrano et al. analyzing the efficacy of Pfizer-BioNTech mRNA vaccination evidenced only five cases of MIS-C in adolescents who have received two doses of vaccine (6). Additionally, other few isolated case reports have been published, including that of a previously vaccinated adolescent patient with sickle cell disease (20). None of the cases reported in literature required intensive care support, thus suggesting a potential protection against life-threatening MIS-C in vaccinated people (6, 20). The low incidence of MIS-C in vaccinated people, together with the reduced spectrum of severity observed significantly support the utility of anti-SARS-CoV-2 vaccination in children and adolescents, although vaccine hesitancy among caregivers is still consistent (21). As the clinical phenotype that we reported in our patient presents peculiar findings, including the prominent hepatic and pancreatic involvement [described in few MIS-C patients (4)] and the absence of a significant cardiovascular impairment, it is possible to hypothesize that the different immunologic background of vaccinated patients could also influence the clinical phenotype of MIS-C. To this regard, the different kinetic of the pulmonary and hepato-pancreatic picture in response to IVIG could be attributed to the secondary activation of the adaptive immunity following the initial cytokine release by innate immune cells.

Another relevant question arising after the introduction of anti-SARS-CoV-2 vaccine in adolescents and, recently, in children aged 5–11 years, is outline the role of the anti-SARS-CoV-2 serologic testing and its correct interpretation to discriminate the immune response to vaccination from the clinical or subclinical infection. Notably, the current platforms to determine prior infection with SARS-CoV-2 rely heavily on the detection of anti-N IgG, and may have decreased sensitivity among the pediatric population. As the case definition of MIS-C is extremely broad and overlaps with other hyperinflammatory conditions, when MIS-C is suspected in an immunized patient, serologic testing not restricted to anti-spike antibodies should be performed (22).

To conclude, it is worth to highlight that the diagnostic and therapeutic approach to the described case presents several areas of uncertainty deriving from the rapidly evolving epidemiological situation, rate of immunization coverage, and update of recommendations. For all these reasons, our aim is to stimulate a debate among experts on the opportunity to update the definition of MIS-C in order to achieve an early diagnosis and stratify the risk for adverse outcomes.

## Data Availability Statement

The original contributions presented in the study are included in the article/supplementary material, further inquiries can be directed to the corresponding author.

## Ethics Statement

Written informed consent was obtained from the minor(s)’ legal guardian/next of kin for the publication of any potentially identifiable images or data included in this article.

## Author Contributions

RC and GC conceptualized the work. GC, ES, AO, and AB collected the clinical data and drafted the manuscript. PC, MB, DP, and RC supervised the data collection and critically reviewed the manuscript. All authors contributed to the article and approved the submitted version.

## Conflict of Interest

The authors declare that the research was conducted in the absence of any commercial or financial relationships that could be construed as a potential conflict of interest.

## Publisher’s Note

All claims expressed in this article are solely those of the authors and do not necessarily represent those of their affiliated organizations, or those of the publisher, the editors and the reviewers. Any product that may be evaluated in this article, or claim that may be made by its manufacturer, is not guaranteed or endorsed by the publisher.
